# Oxford Cognitive Screen – Brazilian Portuguese version (OCS-Br): assessment of vascular cognitive impairment

**DOI:** 10.1055/s-0045-1806919

**Published:** 2025-05-20

**Authors:** Claudia Cristina Ferreira Ramos, Marcelo Vilela Machado João, Caroline Suemi Ogusuku, Sonia Maria Dozzi Brucki

**Affiliations:** 1 Universidade de São Paulo, Hospital das Clínicas, Grupo de Neurologia Cognitiva e do Comportamento, São Paulo SP, Brazil.; 2 Universidade Municipal de São Caetano do Sul, Especialidade de Medicina de Família e Comunidade, São Caetano do Sul SP, Brazil.

**Keywords:** Stroke, Mental Status and Dementia Tests, Cognitive Dysfunction, Dementia, Vascular

## Abstract

**Background:**

Cognitive impairment is prevalent in stroke patients and is rarely diagnosed. Cognitive deficits involving language functions, praxis, visuospatial and visuoconstructive skills, as well as memory, are prominent. The cognitive assessment tests available do not address some specific characteristics of stroke patients and present essential limitations concerning the most compromised cognitive domains.

**Objective:**

To determine the performance profile of the Oxford Cognitive Screen – Brazilian Portuguese version (OCS-Br) in cognitively-healthy individuals and to evaluate its ability to screen for cognitive impairment in individuals after ischemic stroke.

**Methods:**

We conducted an observational and descriptive study with cognitively-healthy individuals and patients with a history of stroke. The healthy individuals were recruited at the Neurology Clinic of the Outpatient Center of Universidade de São Caetano do Sul and the João Castaldelli Integrated Center for Health and Education for the Elderly, in the city of São Caetano do Sul, state of São Paulo. The stroke patients were recruited at the same Neurology Clinic and among subjects referred from Hospital Municipal de Emergências Albert Sabin and admitted to the Stroke Unit of Hospital Santa Marcelina, in the city of São Paulo, from September 2021 to July 2023.

**Results:**

The study included 108 participants, 50 (46.3%) in the stroke group and 58 (53.7%) in the healthy group. When comparing the OCS-Br scores between the groups, we found a significant difference in writing tasks, executive functions (attention, change of strategy), and memory.

**Conclusion:**

Our results show the need for adequate monitoring and rehabilitation of poststroke patients. The advantages of the OCS-Br are: its focus on specific cognitive aspects of stroke, such as visual inattention and visual field testing; the assessment of patients with aphasia and visual impairment; and its prognostic value to predict long-term functioning.

## INTRODUCTION

Globally, stroke was the second leading cause of death in 2019, with 12.2 million cases, 101 million prevalent cases, and 55 million deaths. In Brazil, in 2020, it was responsible for 99,010 deaths, remaining the second cause of death in the country, being the most significant cause of disability in the population in the age group over 50 years, accounting for 10% of total deaths, 32.6% of deaths due to vascular causes, and 40% of early functional disabilities. 1 2 3

Most studies on cognitive dysfunction after stroke use the Mini-Mental State Examination or tests with language biases. We needed a brief examination that could evaluate different domains and enable us to suspect cognitive impairment.

The Oxford Cognitive Screen (OCS) was created to measure common problems after stroke and minimize confusion due to aphasia and neglect. The test consists of 10 items encompassing several cognitive domains, such as attention and executive function, memory, language, praxis, number processing, and visuospatial function. It was designed to be performed with one hand to reduce the impact of upper limb motor weakness. It can be applied in 15 minutes, performed at the bedside, and used in a relatively acute phase of a stroke (3 days after onset). 4 5 The OCS was translated to Brazilian Portuguese (OCS – Brazilian Portuguese version, OCS-Br), and minimal cultural modifications were made to certain items and selected subitems. 5

The objectives of the current study were to determine the performance profile of the OCS-Br in cognitively-healthy individuals in a sample with a wide variation in terms of age and schooling, and to evaluate its ability to screen for cognitive impairment in individuals after ischemic stroke.

## METHODS

We conducted an observational and descriptive study with cognitively-healthy individuals and patients with a history of stroke from the Brazilian Unified Health System (Sistema Único de Saúde, SUS, in Portuguese) from September 2021 to July 2023.

The healthy individuals were recruited at the Neurology Clinic of the Outpatient Center of Universidade de São Caetano do Sul and the João Castaldelli Integrated Center for Health and Education for the Elderly, in the city of São Caetano do Sul, state of São Paulo. The stroke patients were recruited at the same Neurology Clinic and among subjects referred from Hospital Municipal de Emergências Albert Sabin and admitted to the Stroke Unit of Hospital Santa Marcelina, in the city of São Paulo.

The following scales were applied: Geriatric Depression Scale (GDS), 6 Cut Down, Annoyed by Criticism, Guilty, and Eye-Opener (CAGE), 7 Functional Activities Questionnaire (FAQ), 8 Mini-Mental State Examination (MMSE), 9 Montreal Cognitive Assessment (MoCA), 10 and Brief Cognitive Screening Battery (BCSB). 11

We applied the OCS-Br 5 to both groups. It can be applied in 15 minutes at the bedside and was used in the relatively acute phase of a stroke (3 days after onset). The OCS-Br is composed of 10 items which aim to detail deficits in specialized functions, such as language (aphasia), intentional movements (apraxia) or categorical recognition (agnosia), executive function, number processing, visuospatial function, memory, and attention.

In addition, we applied the Trial of ORG 10172 in Acute Stroke Treatment (TOAST) classification 12 and the modified Rankin Scale 13 (mRS) to the stroke group.

The classification was based on the results of the aforementioned tests, and the subjects were divided into cognitively-healthy (no cognitive deficit and no functional impairment), cognitive impairment without dementia (CIWD), and cognitive impairment with dementia (DEM). ( Table 1 ).

**Table 1:** Classification according to the scores on the tests applied to the study sample

Scale	Cognitively-healthy	CIWD	DEM
MMSE	Above the median by schooling and	< 1.5 standard deviations per level of schooling and/or	< 1.5 standard deviations per level of schooling and/or
BCSB	> 7 points and	< 7 points and/or	< 6 points and/or
BCSB–animal category	> 9 for illiterate and < 12 for literate and	< 9 for illiterate and < 12 for literate and	< 9 for illiterate and < 12 for literate and
FAQ	0–2 points	≤ 5 points	> 5 points

Abbreviations: BCSB, Brief Cognitive Screening Battery; CIWD, cognitive impairment without dementia; DEM, cognitive impairment with dementia; FAQ, Functional Activities Questionnaire; MMSE, Mini-Mental State Examination.

Source: Brucki et al., 9 Nitrini et al., 11 and Pfeffer et al. 8

The general inclusion criteria were age between 50 and 90 years, native Brazilian Portuguese speakers, and scores lower than 6 on the GDS. 6 Subjects with decompensated systemic diseases or previous neurological diseases were not included in the control group. The stroke group met the general inclusion criteria, with ischemic stroke confirmed by neuroimaging and evaluation up to 3 months after the stroke.

The exclusion criteria were individuals with a history of stroke without imaging evidence; individuals with previous neurological disease, except stroke that causes speech and language problems, reading difficulties, and/or learning difficulties; and scores higher than 1 point on the CAGE.

The study was approved by the Ethics on Research Committee of Universidade de of São Paulo (USP, under number 3,732,143). All participants signed the free and informed consent form.

## RESULTS

We included 108 participants: 50 (46.3%) in the stroke group and 58 (53.7%) in the healthy group. Table 2 presents the demographic data of the participants. No differences were observed between the groups concerning sex, age, or years of schooling. Based on the median values for age and years of schooling, we cut the database and compared both to check for any statistical differences, which were not found ( *p* < 0.572). This is due to the balance between age and education in the dataset.

**Table 2:** Demographic data according to group

Variable		Stroke group	Healthy group	*p* a
		(N = 50)	(N = 58)	
**Sex**	Female: n (%)	33 (66%)	43 (74.1%)	0.238
	Male: n (%)	17 (34%)	15 (25.9%)	
**Age (in years)**	mean ± SD	67.18 ± 7.8	69.26 ± 7.9	0.271
	median (IQR)	66 (61–73)	68 (63–75)	
**Years of schooling**	mean ± SD	8.56 ± 4.31	9.19 ± 4.09	0.490
	median (IQR)	9 (4–11)	10 (5–11)	

Abbreviations: IQR, interquartile range; SD, standard deviation.

Notes: Statistical significance: *p* < 0.05; ^a^ Mann-Whitney test.

We observed differences between the groups in terms of general cognition, functionality, and mood, with the stroke group presenting significantly lower scores on the GDS, FAQ, MMSE, and MoCA. Regarding the BCSB, we observed no differences between the groups in any of the tasks ( Table 3 ).

**Table 3:** Analysis of the scores on cognitive and mood assessments according to test and group

Test	Stroke group: median (IQR)	Healthy group: median (IQR)	*p* a
	(N = 50)	(N = 58)	
**CAGE**	0	0	0.286
**GDS**	4 (2–7)	2 (1–4)	**0.001**
**FAQ**	2.5 (0–12)	0 (0–2)	**< 0.001**
**MMSE**	25 (21–28)	28 (25–29)	**0.001**
**MoCA**	20 (15–24)	23 (21–25)	**0.003**
**BCSB:**			
*Picture naming*	10 (10–10)	10 (10–10)	0.654
*Perception*	10 (10–10)	10 (10–10)	0.654
*Incidental memory*	5 (4–6)	5 (4–6)	0.799
*Immediate memory*	7 (6–8)	7 (6–8)	0.642
*learning*	8 (6–9)	8 (7–9)	0.321
*Verbal fluency*	12 (10–15)	13 (10–17)	0.325
*Clock drawing*	8 (5–10)	9 (4–10)	0.482
*dealyed recall*	7 (6–9)	7 (6–9)	0.917
*Recognition*	9 (8–10)	10 (9–10)	0.075

Abbreviations: BCSB, Brief Cognitive Screening Battery; CAGE, Cut Down, Annoyed by Criticism, Guilty, and Eye-Opener; FAQ, Functional Activities Questionnaire; GDS, Geriatric Depression Scale; IQR, interquartile range; MMSE, Mini-Mental State Examination; MoCA, Montreal Cognitive Assessment.

Notes: The values in bold indicate statistical significance ( *p* < 0.05); ^a^ Mann-Whitney test.

The mean time since the ictus was of 18.72(±31.97) days, with a minimum of 3 days, a maximum of 90 days, and a median of 4 days. The date of the ictus was only ignored for 1 patient. Regarding the TOAST classification, 10% of the subjects presented atherosclerosis of large arteries, 6%, cardioembolic stroke, 8%, occlusion of small arteries, 2%, infarctions due to other etiologies, and 22%, infarcts of undetermined etiology. Most patients presented mild disability (considering mRS scores of 0, 1, or 2). Unfortunately, 52% of the patients lacked TOAST and mRS assessments.

When comparing the cognition and the TOAST classifications, we observed changes in DEM patients, such as atherosclerosis of large arteries (36.4%), cardioembolic stroke (18.2%), and infarctions of undetermined origin (36.4%). When comparing the mRS scores, there was variation among DEM patients, with most presenting mild disability (36.4%) ( Table 4 ).

**Table 4:** Percentage of TOAST classification and mRS according to cognition

Classification/Scale		Cognitively-healthy: n (%)	CIWD: n (%)	DEM: n (%)
**TOAST classification (N = 24)**	Large-artery atherosclerosis	1 (20%)	0 (0.0%)	4 (36.4%)
	Cardioembolism	1 (20%)	0 (0.0%)	2 (18.2%)
	Small-vessel occlusion	1 (20%)	2 (25%)	1 (9%)
	Stroke of other determined etiology	0 (0.0%)	1 (12.5%)	0 (0.0%)
	Stroke of undetermined etiology	2 (40%)	5 (62.5%)	4 (36.4%)
	Total	5 (100%)	8 (100%)	11 (100%)
**mRS (N = 24)**	No significant disability	1 (20%)	0 (0.0%)	1 (9.1%)
	Mild disability	2 (40%)	8 (100%)	3 (27.2%)
	Moderate disability	2 (40%)	0 (0.0%)	4 (36.4%)
	Moderate/Severe disability	0 (0.0%)	0 (0.0%)	2 (18.2%)
	Serious disability	0 (0.0%)	0 (0.0%)	1 (9.1%)
	Total	5 (100%)	8 (100%)	11 (100%)

Abbreviations: BCSB, Brief Cognitive Screening Battery; CIWD, cognitive impairment without dementia; DEM, cognitive impairment with dementia; FAQ, Functional Activities Questionnaire; MMSE, Mini-Mental State Examination; mRS, modified Rankin Scale; TOAST, Trial of ORG 10172 in Acute Stroke Treatment.

When comparing the cognition status according to the TOAST classification, we observed differences in cognition among the groups, with most DEM patients classified with atherosclerosis of large arteries as the stroke etiology (36.4%) ( Table 4 ).

We found statistically significant differences in OCS-Br tasks between the healthy and stroke groups, such as writing numbers, correct hearts, hearts – right failure, free recall, recognition, triangles, and alternate trails, with impairment mainly in executive functions and memory ( Table 5 ).

**Table 5:** Analysis of the median scores on OCS-Br domains of the studygroups

OCS-Br domain	Stroke group: median (IQR)	Healthy group: median (IQR)	*p* a
	(N = 50)	(N = 58)	
**Picture naming**	4 (3–4)	4 (3–4)	0.912
**Semantics**	3 (3–3)	3 (3–3)	0.729
**Orientation**	4 (4–4)	4 (4–4)	0.077
**Visual fields**	4 (4–4)	4 (4–4)	0.149
**Sentence reading**	15 (14–15)	15 (14–15)	0.864
**Number writing**	3 (3–3)	3 (3–3)	**0.023**
**Calculation**	3 (2–4)	3 (3–4)	0.761
**Broken hearts**	46 (41–49)	48 (47–49)	**0.005**
**Hearts – Left**	0 (0–2)	0 (0–1)	0.092
**Hearts – Right**	0 (0–2)	0 (0–1)	**0.021**
**Imitation**	12 (8–12)	12 (9–12)	0.149
**Free recall**	0 (0–2)	2 (1–3)	**0.001**
**Recognition**	3 (2–4)	4 (3–4)	**0.004**
**Episodic memory**	4 (3–4)	4 (3–4)	0.588
**Circles**	6 (2–6)	6 (5–6)	0.062
**Circles –in seconds**	22 (15–33)	18 (13–30)	0.152
**Triangles**	6 (3–6)	6 (6–6)	**0.016**
**Triangles – in seconds**	20 (15–31)	16 (12–25)	0.057
**Alternate tracks**	7 (2–13)	11 (8–13)	**0.035**
**Alternate tracks – in seconds**	45 (35–64)	52 (40–63)	0.310

Abbreviations: IQR, interquartile range; OCS-Br, Oxford Cognitive Screen – Brazilian Portuguese version.

Notes: The values in bold indicate statistical significance ( *p* < 0.05); ^a^ Mann-Whitney test.

When comparing the OCS-Br scores in terms of the age cutoff (< 65 and > 65 years) and the schooling cutoff of 4 years, we did not find statistically significant differences between the groups. However, when comparing the OCS-Br scores in terms of the the grouped mRS scores (mild and severe disability), we found statistically significant differences between the groups regarding orientation ( *p* < 0.015) and triangles ( *p* < 0.023).

The comparison of OCS-Br scores in terms of the cognition classification was carried out considering the subgroups of cognitively-healthy, CIWD, and DEM subjects, with a statistically significant result regarding orientation, the visual field test, free recall, recognition, and circles (in seconds) ( Table 6 ).

**Table 6:** Analysis of the median scores on OCS-Br domains separated by the cognition classification

OCS-Br domain	Cognitively-healthy: median (IQR)	CIWD/DEM: median (IQR)	*p* a
	N = 38	N = 70	
**Picture naming**	4 (4–4)	4 (3–4)	0.075
**Semantics**	3 (3–3)	3 (3–3)	0.192
**Orientation**	4 (4–4)	4 (4–4)	**0.013**
**Visual fields**	4 (4–4)	4 (4–4)	**0.045**
**Sentence reading**	15 (14–15)	15 (14–15)	0.802
**Number writing**	3 (3–3)	3 (3–3)	0.062
**Calculation**	4 (3–4)	3 (2–4)	0.111
**Broken hearts**	48 (46–49)	47 (43–49)	0.229
**Hearts – Left**	0 (0–1)	0 (0–2)	0.457
**Hearts – Right**	0 (0–1)	0 (0–1)	0.794
**Imitation**	12 (10–12)	12 (9–12)	0.488
**Free recall**	2 (1–3)	1 (0–2)	**0.002**
**Recognition**	4 (3–4)	4 (2–4)	**0.010**
**Episodic memory**	4 (4–4)	4 (3–4)	0.062
**Circles**	6 (5–6)	6 (3–6)	0.224
**Circles –in seconds**	15 (13–25)	23 (17–37)	**0.008**
**Triangles**	6 (6–6)	6 (3–6)	0.063
**Triangles – in seconds**	17 (14–24)	20 (13–31)	0.327
**Alternate tracks**	12 (7–13)	9 (4–13)	0.070
**Alternate tracks – in seconds**	45 (34–58)	54 (40–66)	0.069

Abbreviations: CIWD, cognitive impairment without dementia; DEM, cognitive impairment with dementia; IQR, interquartile range; OCS-Br, Oxford Cognitive Screen – Brazilian Portuguese version.

Notes: The values in bold indicate statistical significance ( *p* < 0.05); ^a^ Mann-Whitney test.

## DISCUSSION

In summary, we observed that the OCS-Br was not influenced by age and years of schooling, distinguishing the control and stroke groups regarding the subitems of number writing, hearts, free recall and recognition, triangles, and alternating trails.

The results differed between the groups according to the mRS in the subitems that evaluate orientation and processing velocity (orientation and triangles in seconds). In differentiating the groups with observed differences in orientation, visual field, memory, and processing velocity.

Care guidelines for stroke patients recommend identifying cognitive deficits soon after the onset of symptoms to plan the most appropriate rehabilitation program, since cognitive impairment is a predictor of poor long-term prognosis. A brief bedside test is necessary to evaluate these patients. Our evaluation was performed on average 18.72 days after the ictus, falling within the recommended range. The OCS was created to measure common problems after stroke and minimize bias due to aphasia or neglect.

In the current study, we observed similar values for each OCS-Br task compared to the original tool, confirming the comparability of the Brazilian Portuguese version. We found a statistical difference among the groups in the tasks of writing numbers, correct hearts, hearts—right failure, free recall, recognition, triangles, and alternating trails.

Kong et al. 14 (2016) validated the Hong Kong version of the OCS (HK-OCS), with the control group performing similarly to ours. According to the authors, 14 male patients were the most affected, both in the stroke group (61%) and in the healthy/control group (57%). Regarding schooling, the patients analyzed by the authors mostly had secondary education (46% in the stroke group and 56% in the control group). Most HK-OCS subtests correlated significantly with subtests that assess cognitive domains like other assessment tools, except for verbal memory (recall and recognition), episodic memory, and the heart neglect test. The best predictors of functional outcomes were specific subtests of the HK-OCS, including semantics, episodic memory, number writing, and orientation.

In the current study, we observed a prevalence of females in the stroke and the control groups, with similar ages and years of schooling. While the MMSE and MoCA scores showed significant differences, there were no disparities in the BCSB scores. Within the OCS-Br assessment, we found differences between the groups in tasks such as writing numbers, free recall, and recognition, while the semantic classification remained consistent across both groups.

Humphreys et al. 15 (2017) validated an OCS-Plus version specifically designed for low-literacy environments. The OCS-Plus assesses cognition regarding physical and mental health, age, schooling, and alcohol consumption. Given the prevalent low levels of schooling in Brazil, 16 this version could be valuable for the Brazilian population. In the present study, 45.4% of the patients had up to 8 years of schooling.

The median values for the years of schooling were of 9 years for the stroke group and of 10 years for the control group, which is in line with data from Instituto Brasileiro de Geografia e Estatística 16 (IBGE, 2022) data, which reported an average of 9.9 years of schooling.

It is worth considering that administering all the scales used in the present study makes the assessment comprehensive, likely encouraging greater patient cooperation. A concise evaluation, or one with minimal linguistic content, as proposed in the OCS-Plus, may benefit the assessment before these patients are discharged from the hospital. We observed that 8 patients did not fully fill out the OCS-Br; 3 mentioned they lacked glasses and could not complete the drawing section, while the rest did not provide a reason.

Murphy et al. 17 (2023) evaluated 316 poststroke patients, conducting a critical review and independent validation of the OCS to understand why patients fail to complete it. In total, 29.8% of their sample could not complete at least 1 subtest. The most common incomplete subtests were the broken heart test and the trail test, with almost one in five patients unable to complete them. The most frequently mentioned reasons were impaired upper limb function, inability to “pass” the practice tracks, fatigue, or the patient's decision to discontinue.

In the present study, there was no statistical significance regarding the OCS-Br items; however, we could observe that the participants presented the most significant difficulties or deficits in the following areas: naming, semantic notation, visual field test, sentence reading, calculations, hearts – left failure, gesture imitation, episodic memory, circles, circles in seconds, triangles in seconds, and alternating trails in seconds.

Our data suggest that the OCS-Br can distinguish between disorders across different cognitive domains and identify impaired and preserved functions. Identifying these cognitive deficits early on contributes to enhancing the success of rehabilitation tailored to the specific affected domains. One crucial aspect is that the OCS detects cognitive deficits frequently associated with stroke that are not captured by other screening tools, such as the MMSE and MoCA.

It is important to emphasize the limitations of using tests originally designed for dementia in stroke populations. Studies 18 have shown greater sensitivity of the OCS compared to the MMSE, with a much higher incidence of cognitive impairment detected using the OCS (91% of impairment in at least 1 domain) than the MoCA (35%). Evidence also indicates better performance of stroke patients in OCS subitems compared to the MoCA 19 and greater sensitivity of the MoCA compared to the MMSE. 20 Overall, it appears that cognitive deficits after stroke are more effectively detected by tools designed specifically for poststroke assessment than by tools originally intended for dementia. 18

Jacinto et al. 21 (2014) investigated the effectiveness of screening instruments for cognitive impairment. The FAQ exhibited the highest sensitivity and specificity among all instruments used in cognitive assessment. The most significant specificity (of 93.5%) was observed in the combination of the FAQ with the verbal fluency test in the animal category. In the current study, the cognitive screening did not reveal significant differences between the groups across various items. However, lower rates were noted concerning recognition and learning.

The data obtained from the OCS-Br aligns with the rates obtained from the mRS scores. According to the mRS scores, in our sample, 26% of the respondents did not exhibit any significant disability. In comparison, 12% displayed mild disability, suggesting a favorable prognosis and the detection of lower levels of cognitive impairment among participants after stroke. Consequently, 38% of the patients interviewed could perform all customary tasks and activities or prior activities and, perhaps most importantly, could attend to their own needs. One may contemplate the extent to which this condition serves as a motivation for improved treatment outcomes and facilitates relationships with close acquaintances.

Based on the TOAST classification, we determined that 10% of respondents in the current study exhibited large artery atherosclerosis, while 8% presented small artery occlusion. However, a limitation of the TOAST classification is its categorization of an undetermined cause of heart attacks, and in the present study, we observed that this category accounted for 22% of our sample.

More than half of stroke survivors meet the criteria for poststroke neurocognitive disorders, and these cognitive challenges strongly correlate with a decline in quality of life and functional independence over the long term. The results of the present study underscore the need for adequate monitoring and rehabilitation of poststroke patients. Numerous organizations advocate for routine screening for cognitive impairments during the acute, subacute, and chronic stages of stroke recovery. Additionally, current practice standards dictate that all stroke survivors undergo cognitive assessments following the event. Although the MoCA is effective in screening for mild cognitive impairment, its application in stroke settings is hampered by several limitations.

During the review of the present work and patient recruitment, we noted several advantages of the OCS-Br, including its focus on specific cognitive aspects of stroke, such as visual inattention and visual field testing, its inclusivity of patients with aphasia and visual impairment, and its value in the prediction of long-term functioning.

However, it is important to acknowledge the limitations of the OCS-Br, such as the need to define its precise role in planning patient follow-up and the challenge of completing it due to its length compared to shorter instruments such as the MMSE.

These points are crucial, as they suggest potential adaptations and enhancements to the instrument and encourage further research into its sensitivity in the poststroke population.

The positive aspects of the current study were the age- and schooling-matched samples, a sufficient number of stroke patients and controls, and a comprehensive assessment of cognition and functionality. Additionally, patients were monitored over time after the stroke, underscoring the importance of early diagnosis and intervention in rehabilitation.

A limiting factor, however, was the relatively high level of schooling of the sample. Nevertheless, it is worth noting that the population of the preset study was from SUS services, highlighting the need to assess the test in different country regions.

In conclusion, we found statistically significant differences in the performance on the OCS-Br between the stroke and healthy groups concerning scores on the following memory items: number writing, correct hearts, hearts – right failure, free recall, recognition, triangles, and alternating trails. However, there were no statistically significant differences when accounting for age and schooling.

Comparing the cognitively-healthy versus the CIWD and DEM patients, we observed changes in orientation, visual field test, free recall, recognition, and circles in seconds.

We consider the OCS-Br to have a good screening ability for cognitive impairment in individuals with ischemic stroke, as it targets specific cognitive aspects of stroke, such as visual inattention and visual field testing. Additionally, it is inclusive of patients with aphasia and visual impairment and has predictive value for long-term outcomes.

It is important to highlight its potential usability in Brazil, where stroke is the leading cause of disability in the population over the age of 50. Cognitive deficits after stroke are more effectively detected by tools specifically designed for this condition than by tools designed for dementia.

Further studies are necessary, considering a larger sample of patients across different age groups and levels of schooling. Additionally, including patients with acute, subacute, and chronic ischemic brain disease would provide more data regarding this population, thereby offering better support throughout the treatment journey, especially with rehabilitation tailored to each patient's profile.
